# Mitigating the Trade-Off between Non-Radiative Recombination and Charge Transport to Enable Efficient Ternary Organic Solar Cells

**DOI:** 10.3390/ma16165620

**Published:** 2023-08-14

**Authors:** Yexin Zhang, Shuai Yuan, Congyang Zhang, Chenfeng Ding, Congcong Zhang, Hai Xu

**Affiliations:** 1 College of Chemistry and Chemical Engineering, Central South University, South Lushan Road, Changsha 410083, China; 22020168@csu.edu.cn; 2 Jiangsu Key Laboratory for Carbon-Based Functional Materials & Devices, Institute of Functional Nano & Soft Materials (FUNSOM), Soochow University, Suzhou 215123, China; 3 Energy Materials and Surface Sciences Unit (EMSSU), Okinawa Institute of Science and Technology Graduate University (OIST), Okinawa 904-0495, Japan; congyang.zhang@oist.jp (C.Z.); chenfeng.ding@oist.jp (C.D.); 4 Department of General Systems Studies, Graduate School of Arts and Sciences, The University of Tokyo, 3-8-1 Komaba, Meguro-ku, Tokyo 153-8902, Japan; zhang.congcong@mail.u-tokyo.ac.jp

**Keywords:** ternary organic solar cells, charge-transfer state, open circle voltage loss, nonfullerene acceptors

## Abstract

Ternary organic solar cells (OSCs) have attracted intensive studies due to their promising potential for attaining high-performing photovoltaics, whereas there has been an opening challenge in minimizing the open circuit voltage (*V*_oc_) loss while retaining the optimal carrier extraction in the multiple mixture absorbers. Here, we systemically investigate a ternary absorber comprised of two acceptors and a donor, in which the resultant *V*_oc_ and fill factor are varied and determined by the ratios of acceptor components as a result of the unbalance of non-radiative recombination rates and charge transport. The transient absorption spectroscopy and electroluminescence techniques verify two distinguishable charge-transfer (CT) states in the ternary absorber, and the mismatch of non-radiative recombination rates of those two CT states is demonstrated to be associated with the *V*_oc_ deficit, whilst the high-emissive acceptor molecule delivers inferior electron mobility, resulting in poor charge transport and a subpar fill factor. These findings enable us to optimize the mixture configuration for attaining the maximal-performing devices. Our results not only provide insight into maximizing the photovoltage of organic solar cells but can also motivate researchers to further unravel the photophysical mechanisms underlying the intermolecular electronic states of organic semiconductors.

## 1. Introduction

The advancements in organic solar cells (OSCs) are recasting the landscape of renewable energy technology due to their unique merits in lightweight design, flexibility, and cost-effectiveness [1]. Those advantages support great interest in the future commercialization of OSCs; considering the facile solution-processing of the organic active layer, the scalable organic solar module with ideal efficiency is expected to appear in the near future. However, cutting-edge OSCs are still limited by subpar stability and low power conversion efficiency (PCE) compared to advanced photovoltaic technologies such as crystalline silicon, GaAs, CdTe, and the new-born perovskite solar cells [2]. However, the predominate bottleneck accounting for the lagging PCE is broadly attributed to low fill factor and high open circuit voltage (*V*_oc_) losses, which leads to a large mismatching between devices’ open circuit voltages and the corresponding photon energy (E_g_/e) at the long-wavelength onset of their absorption. The low-dielectric ($\varepsilon_{r}$ = 3–4) properties of organic semiconductors afford their unique electronic intermediate states, known as the charge-transfer (CT) state, that dominates the charge separation and transport at the interface of donor and acceptor units, and in turn, are closely related to devices *V*_oc_ [3]. Ternary OSCs appear as an alternative approach to enhance the device performance, bringing the understanding of CT states to a new stage. The complex donor–acceptor mixture and the ensuing multiple electronic intermediate states accompanied by small *V*_oc_ losses of ternary OSCs exemplify an advanced paradigm to study the underpinning relations between charge transport and energy-losing factors. Recently, Gao et al. unraveled the underpinning factors that determine the energy loss of OSCs, in which a highly emissive ternary mixture, accompanied by the small energy mismatch among the CT states as well as superb miscibility between the guest component and the low gap component, are suggested to afford minimal *V*_oc_ loss [4,5,6]. However, the relevant research remains in its infancy, calling for a comprehensive understanding to guide future progresses.

Herein, we center around a ternary absorber system composed of the PBDB-T donor and two non-fullerene acceptors regarding the ITIC-TH molecule and a BDT-IC derivative to reveal the ensemble characteristics of multiple CT states and charge transport in a single entity. Electroluminescence (EL) approach points out two distinct CT states in such ternary OSCs; meanwhile, the EL efficiencies (*EQE_EL_*) of CT states are considerably different, implying the largely varied non-radiative recombination. This property leads to a set of tunable *V*_oc_ in devices consisting of different donor and acceptor ratios, whereas the absorber mixtures featuring maximal *V*_oc_ are limited by struggling charge separation. On the aforementioned discoveries, we balance those contributing factors to allow maximal power conversion efficiency. Our finds represent a new view for achieving high PCE in OSCs based on the ternary absorber, which would take full advantage of organic materials to realize advanced optoelectronic devices.

## 2. Results and Discussion

**Figure 1:**
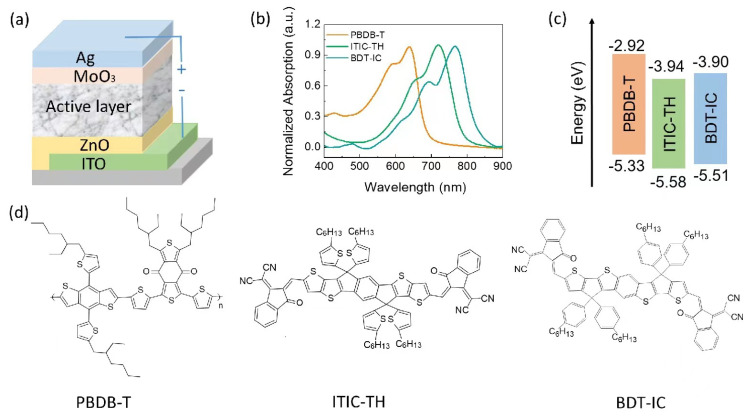
(**a**) Schematic representation of the device stacks of OSCs. (**b**) The normalized UV-vis absorption spectra of bare PBDB-T, ITIC-TH, and BDT-IC films. (**c**,**d**), Energy level alignment (**c**) and chemical diagram of the materials (**d**) in the active layer.

**Figure 2:**
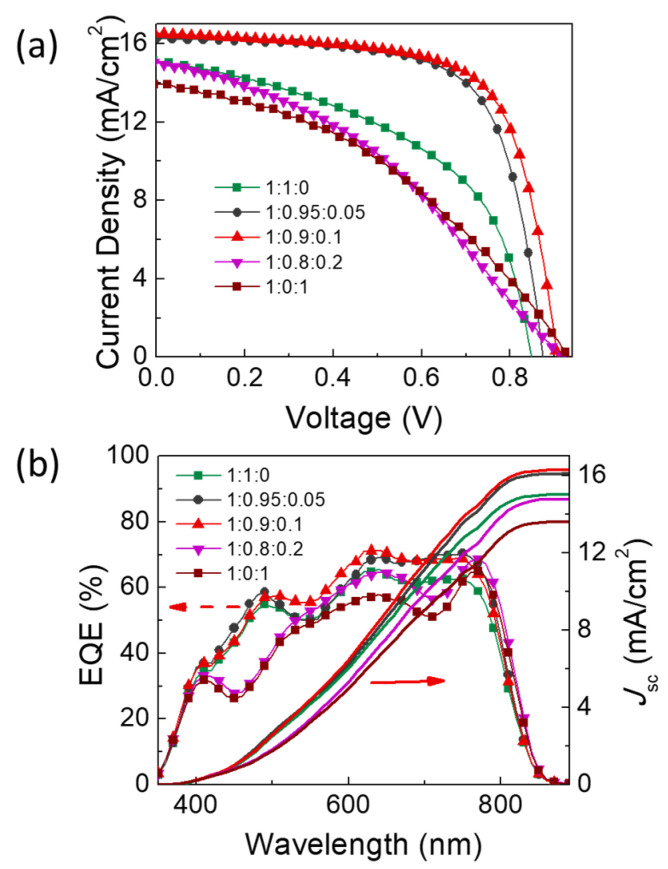
(**a**) *J-V* curves of OSCs consisting of various compositional ratios under the illumination of AM1.5 G. (**b**) The corresponding EQE spectra.

The device stacks, complementary absorption properties of absorbers, energy level alignment, and chemical architectures of donor and acceptors are shown in Figure 1. The structural features and topologic properties, as well as the miscibility of the ternary mixture films, are characterized using XRD, SEM, and atomic force microscope (AFM) images (Figures S1–S3). Figure 2a presents the current density–voltage (*J-V*) curves of ternary blend OSCs with different BDT-IC weight content under AM 1.5 illumination with a light intensity of 100 mW/cm^2^. Table 1 summarizes the detailed photovoltaic parameters for those devices. The PBDB-T: ITIC-TH and PBDB-T: BDT-IC were regarded as reference devices. The PBDB-T: ITIC-TH binary device delivered an average PCE of 6.47% with *V*_oc_ of 0.82 V, *J*_sc_ of 15.42 mA/cm^2^, and FF of 0.49. Another standard device, PBDB-T: BDT-IC, exhibited an average PCE of 5.74%, with a higher *V*_oc_ of 0.92 V, *J*_sc_ of 13.71 mA/cm^2^ and low FF of 0.45. With the third component BDT-IC of 10 wt% in the total acceptor, the ternary blend OSCs delivered an optimized PCE attained 10.25%, whose *V*_oc_ gradually increased to 0.90 V, *J*_sc_ raised to 16.52 mA/cm^2^, and FF exhibited to a limited value of 0.69. However, device performance could be affected by excess BDT-IC. The *V*_oc_ in our organic solar cells is not simply determined by the HOMO level of donor material and LUMO level in the traditional multi-blend systems, whose *V*_oc_ is pinned at the lowest *V*_oc_ in the corresponding binary system. It is the certification that there coexist two CT states in our ternary blend system. The EQE curves of ternary OSCs are plotted in Figure 2b. The admixture of 10 wt% BDT-IC attained a maximum EQE value of 71.43%. The calculated integral *J*_sc_ from EQE is 15.00 mA/cm^2^, 16.09 mA/cm^2^, 16.29 mA/cm^2^, 14.78 mA/cm^2^, and 13.61 mA/cm^2^, respectively, concordance with the changing of the experiment.

**Figure 3:**
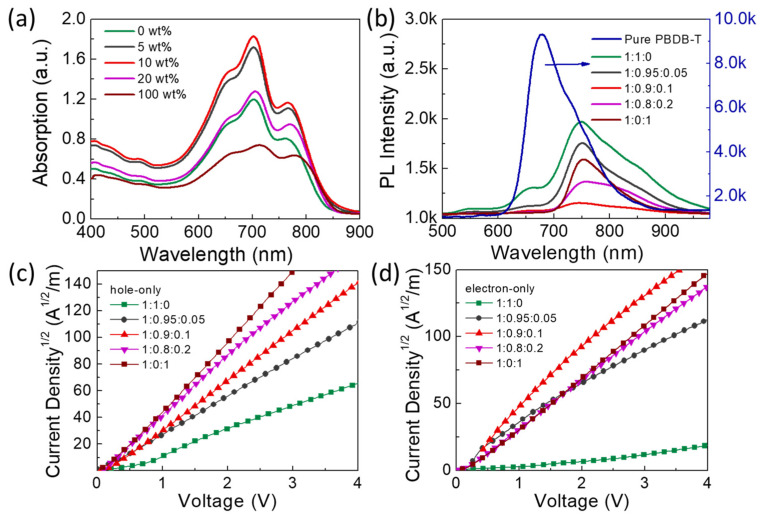
(**a**) UV-vis absorption spectra. (**b**) The PL spectra of bare PBDB-T and blend films excited at 450 nm. (**c**,**d**) Hole (**c**) and electron (**d**) mobility of absorbers with varied BDT-IC ratios.

The blend films’ absorption spectra with different BDT-IC contents were measured and summarized in Figure 3a. It demonstrates that incorporation of BDT-IC may expand the photon harvesting range of films to 840 nm compared to the films without BDT-IC, in accordance with the trend of the corresponding resultant *V*_oc_ of their devices listed in Table 1 that the band gap of blend films becomes narrow with narrowband gap material BDT-IC. The photoluminescence (PL) of films is an efficient technique to identify exciton diffusion and separation dynamics. The steady-state PL spectra of pure polymer PBDB-T and blend film are depicted in Figure 3b, which were tested three times and averaged. The steady-state PL quenching efficiency (∆PL) is defined as the PL intensity of the polymer: acceptor blends relative to that of the neat polymer film [7]. Interestingly, ∆PL in the blend system are 43%, 53%, 83%, 73%, and 61%, respectively, which is a coincidence with the variety of PLQY listed in Table S2, Supplementary Materials. A higher ∆PL indicates the efficiency of exciton diffusion and dissociation at the interface of donor and acceptor. To provide more credible evidence for better performance, ternary OSCs, hole- and electron-only devices were measured in the dark. The mobilities of the hole and electron were calculated using the space charge limited current (SCLC) method defined by the Mott–Gurney law [8]. Figure 3c,d displayed the *J^*0.5*^-V* curves with corresponding active layers. Detailed results are listed in Table 1. The hole ($\mu_{h}$) and electron mobility ($\mu_{e}$) are both enhanced with the third component. Moreover, the resultant $\mu_{h}/\mu_{e}$ reaches 1.04 in the optimized ternary OSCs, indicating a balance carrier transport, thus resulting in higher FF and *J*_sc_ in ternary OSCs.

**Figure 4:**
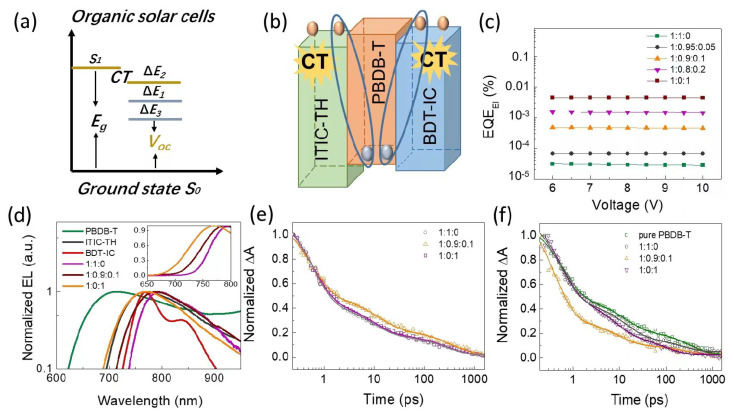
(**a**) Energy loss in OSCs. (**b**) Coexistence of CT states at the interfaces of donor and acceptor components. (**c**) *EQE_EL_* measurements of blend system as a function of the external bias. (**d**) EL spectra of different emitters; the insert is the linear relationship graph of EL spectra. (**e**,**f**) Excited states dynamics of various absorbers, at 1.61 eV (**e**) and 1.26 eV (**f**).

To gain further insight into increased *V*_oc_ and the nature of its high photovoltaic performance in ternary blend systems, we investigated voltage losses in ternary OSCs. Conventionally, the voltage loss (∆E) is defined as the difference between the optical gap of blend solar cells and *V*_oc_. As shown in Figure 4a, it specifically includes three parts [9]:

$$\begin{array}{c} ∆E=E_{g}-qV_{oc}\\ =(E_{g}-qV_{oc}^{SQ})+\left(qV_{oc}^{SQ}-qV_{oc}^{rad}\right)+(qV_{oc}^{rad}-qV_{oc})\\ =(E_{g}-qV_{oc}^{SQ})+q{∆V}_{oc}^{rad,blow\;gap}+q{∆V}_{oc}^{non-rad}\\ ={∆E}_{1}+{∆E}_{2}+{∆E}_{3} \end{array}$$

The first part ${∆E}_{1}=E_{g}-qV_{oc}^{SQ}$ stems from the absorption and emission of all energy above a certain band-gap due to radiative recombination, typically 0.25 eV or above, unavoidably for any solar cells. Here, *E*_g_, defined the difference between the S_1_ state determined by the crossing point of normalized absorption and emission curves and vibrationally relaxed ground state, and ref. [10] is the lowest gap of blend films. $V_{oc}^{SQ}$ is the theoretical maximum voltage limited by the Shockley–Queisser (SQ). The detailed calculation process can be found in Supplementary Materials.

The second part $∆E_{2}=qV_{oc}^{SQ}-qV_{oc}^{rad}$ is caused by absorption below the band gap. $V_{oc}^{rad}$ is the open-circuit voltage when there is only radiative recombination. Unlike the ideal step-wise function absorption of the SQ limit, the absorptance in $V_{oc}^{rad}$ is the actual absorptance originating from the CT state of a solar cell.

The last part of voltage loss $E_{3}=qV_{oc}^{non-rad}$ originates from non-radiative recombination, directed determined by the equation $∆V_{oc}^{non-rad}=kTln(1/{EQE}_{EL})$, where *EQE_EL_* is the external quantum yield when charge carriers are injected into the device in the dark [11]. Defects and impurities performed as recombination centers, leading to non-radiative recombination in real solar cell devices. Low non-radiative voltage loss can be achieved when *EQE_EL_* is enhanced according to the formula listed. The *EQE_EL_* curves were plotted in Figure 4c.

Ordinarily, *V*_oc_ loss in high-performance OSCs is classified into two contents. One of them is large recombination loss owing to the monomolecular and bimolecular recombination during the process of CT state generation, split, and charge transport. Another originates from the CT state formation of energy offset between the S1 state of donor/acceptor materials and the CT state, corresponding to the driving force, which is necessary for photoinduced charge separation in OSCs [12]. Generally, it is recognized that a necessary driving force is needed for efficient charge separation. Hence, it is difficult to reduce driving force and realize fast and efficient charge transfer at the same time. Therefore, we attempt to determine the energy of charge transfer (E_CT_) state of the blend films through electroluminescence measurement to evaluate the driving force in our OSCs. The electroluminescence (EL) spectra of pure ITIC-TH and the CT emission of PBDB-T: ITIC-TH, PBDB-T:ITIC-TH:BDT-IC (1:0.9:0.1), and PBDB-T:BDT-IC are shown in Figure 4d. Compared to the EL spectra of pure materials, the blend films express redshifted emitting arising from the CT state. Significantly, the EL peaks gradually blue shift with blending the third component material BDT-IC of insert in Figure 4d, suggesting the two coexistent CT states in mixing films, the higher CT state of PBDB-T: BDT-IC and the lower CT state of PBDB-T: ITIC-TH, shown in Figure 4b. The two various parallel solar cells, i.e., the higher CT state blend film OSCs with BDT-IC molecule as acceptor, which shows low non-radiative recombination loss and lower CT state OSCs with ITIC-TH as acceptor with high non-radiative recombination loss, lead to a tunable *V*_oc_ in the ternary blend OSCs perform. Comparing the PL and EL spectra, Figures S5–S7 in Supplementary Materials, the different locations of peaks of EL and PL indicate exciton recombination not only come from one state. At the same time, there is no transformation for the location of PL, coincidently with the conclusion that the S_1_ and CT state are approaching [13,14,15,16,17,18]. Meanwhile, the driving force is gradually diminishing with a higher CT state according to the definition, coincidentally with a reduced photocurrent and inferior device performance in the PBDB-T:BDT-IC solar cell.

The question appeared about the charge separation process in this blend with such a negligible driving force. Then, the Femtosecond Transient Absorption Spectroscopy (TAS) was performed to investigate the generation and dissociation dynamics of exciton and polaron in the blend system. Figure 4e,f presents the transient absorption spectra (∆A) as a function of time decay at varied wavelengths in the near-infrared region. Here, the ∆A spectra indicate a photoinduced exciton absorption. Figures S8 and S9, Supplementary Materials, display the TAS of neat PBDB-T, PBDB-T: ITIC-TH, PBDB-T:ITIC-TH:BDB-IC (1:0.9:0.1), PBDB-T:BDB-IC films at different delay time, respectively. The strong positive band centered at 770 nm vanished at the pure PBDB-T TAS due to the CT state (the polymer polarons) at the blend system. Data as a function of time delay were probed at 980 nm ascribed polymer singlet exciton decay.

As shown in Figure 4e,f, time evolution analysis indicates that the dynamics in the ternary system are quite similar to the dynamics process in binary blend OSCs. For the ternary blend system, the polaron decayed lifetime, as shown in Figure 4e, was 263 ps and 322 ps, 271 ps; the large percentage of long-lived polaron favored efficient charge generating and suppressing geminate molecular recombination in the blend system. The singlet exciton decay lifetime of the blend system is 75 ps and 45 ps, while it is about 18 ps in the ternary blend system; the shorter lifetime indicates the rapidity of exciton diffusion and dissociation, depicted in Figure 4f. The pure polymer PBDB-T transient absorption spectrum is used for comparison with a singlet exaction lifetime of 153 ps, revealing that about 88% of exciton exist on the polymer in the ternary blend system are dissociating, and 12% of them are quenching due to the size of the fraction of mixed regions and/or domains. This consequence indicates that the driving force has a small influence on the device’s performance.

The aforementioned results encourage us to perform maximum power point tracking (MPPT) measurements to understand the output stability of the optimal OSCs, as shown in Figure S10. The optimal device shows decent power output, retaining 80% of initial PCE after 20 h.

## 3. Conclusions

The trinary OSCs consisting of a PBDB-T donor and two non-fullerene accepters regarding BDT-IC and ITIC-TH were developed with maximal device performance. We demonstrated a balance between electron and hole mobilities in an optimal mixture with 10 wt% BDT-IC content. It is noteworthy that the PL and EL results highlight the exciton diffusion, exciton dissociation, and non-radiative loss characterized by blending BDT-IC in the binary system. *EQE_EL_* data indicated low non-radiative recombination loss in PBDB-T: BDT-IC based solar cells. Thus, the parallel cells of PBDB-T: BDT-IC and PBDB-T:ITIC-TH affected the total performance of the ternary system cooperatively, and the ternary OSCs deliver tunable *V*_oc_. And *J*_sc_ also increased by blending the narrower band gap material at the same time. Meanwhile, TAS exhibited that the quite small driving force despite, the system, could provide quite a few forces for exciton separation in our ternary OSCs. As a result, our ternary OSCs exhibited a maximum PCE of 10.25%, with *V*_oc_ = 0.90 V, *J*_sc_ = 16.52 mA/cm^2^, and FF = 0.69.

## Figures and Tables

**Table 1:** Summary of OSCs parameters of ternary PBDB-T: ITIC-TH blends with different ratios of BDT-IC under standard AM 1.5 illumination.

Devices	*V*_oc_ (V) ^a^ /MAX	*J*_sc_ (mA/cm^2^) ^a^ /MAX	FF ^a^ /MAX	PCE (%) ^a^ /MAX	$\mu_{h}\times 10^{-4}$ (cm^2^V^−1^s^−1^) ^b^	$\mu_{e}\times 10^{-4}$ (cm^2^V^−1^s^−1^) ^b^	$\mu_{h}/\mu_{e}$
1:1:0	0.80/0.82	15.42/15.93	0.49/0.53	6.47/7.14	1.05	0.152	6.94
1:0.95:0.05	0.85/0.87	15.80/16.29	0.67/0.69	9.23/9.80	2.43	1.86	1.30
1:0.9:0.1	0.88/0.90	16.01/16.52	0.67/0.69	9.96/10.25	4.53	4.36	1.04
1:0.8:0.2	0.89/0.91	15.17/15.57	0.400.41	5.53/5.80	7.11	4.80	1.48
1:0:1	0.90/0.92	13.51/13.61	0.45/0.50	5.74/6.32	9.88	5.34	1.85

^a^ The average data were calculated from ten devices at different ratios. ^b^ Calculated from the space charge-limited current (SCLC) model.

## Data Availability

The data that support the findings of this study are available within the article and its Supplementary Materials.

## Supplementary Materials

The following supporting information can be downloaded at: https://www.mdpi.com/article/10.3390/ma16165620/s1; see the Supplementary Materials for the experimental section, Figures S1–S10, and Tables S1 and S2. References [6,16,17,18] are cited in the Supplementary Materials.
