# Supplementary material for: Mitigating the Trade-Off between Non-Radiative Recombination and Charge Transport to Enable Efficient Ternary Organic Solar Cells
Source: Materials (Basel). 2023 Aug 14;16(16):5620. doi: 10.3390/ma16165620 (PMC10456567; doi:10.3390/ma16165620)
Supplement: Supplementary file 1 [file materials-16-05620-s001.zip › materials-2497986-supplementary.pdf]

Material and Experimental Section:

All starting materials and reagents were purchased from commercial sources: MoO_3 (Acros Organics), DTDCPB, BPhen and TmPyPB (Luminescence Technology Corp.), C70 (SES Research), C_{60} (MER Corp.), PEDOT:PSS (Clevios P VP AI. 4083 (Heraeus)), PBDB-T (1-Material) and Ag (Alfa Aesar), and used without further purification unless otherwise specified. purchased from commercial suppliers: All solvents were dehydrated and syringes used to transfer reagents or solvents were purged with Nitrogen prior to use.

UV-Vis spectra were measured using a Perkin-Elmer Lambda-9 spectrophotometer. The 1H and 13C NMR spectra were collected on a Bruker AV400 and 600 spectrometer in deuterated chloroform solution with TMS as reference. Time of Flight MS – MALDI (TOF) MS were performed on a Bruker Autoflex II / Compass 1.0 from Department of Materials Science and Engineering in Soochow University. Differential scanning calorimetry (DSC) was performed using a TA DSC 2010 unit under nitrogen at a heating rate of 10 oC/min from room temperature to 300 oC. Thermogravimetric analysis (TGA) was performed on a TA SDT 2960 instrument at a heating rate of 10 oC/min from room temperature to 800 oC under nitrogen. Cyclic voltammetry of polymer film was conducted in acetonitrile with 0.1 M of tetrabutylammonium hexafluorophosphate using a scan rate of 100 mV s -1. ITO, Ag/AgCl and Pt mesh were used as working, reference, and counter electrodes, respectively. Atomic force microscopy (AFM) images of the interfacial films were obtained using a Veeco Multimode V instrument. TEM images was recorded on a Tecnai G2 F20 S-TWIN instrument at 200 kV accelerating voltage. Ultraviolet photoelectron spectroscopy was performed on Kratos AXIS ULTRA DLD instrument with UV light source is not monochromatic He I (21.22eV). Grazing incidence x-ray diffraction (GIXD)

characterization of BDT-IC was performed at beamline 7.3.3, Advanced Light Source (ALS), Lawrence Berkeley National Lab (LBNL). X-ray energy was 10 keV and operated in top off mode. The scattering intensity was recorded on a 2D image plate (Pilatus 1M) with a pixel size of 172 m (981 × 1043 pixels). The samples were ~10 mm long in the direction of the beam path, and the detector was located at a distance of 300 mm from the sample center (distance calibrated by AgB reference). The incidence angle was chosen to be 0.16o (above critical angle) for GIXD measurement. OPV samples were prepared on PEDOT:PSS covered Si wafers in a similar manner to the OPV devices. RSAXS was performed at beamline 11.0.1.2 Lawrence Berkeley National Lab. Thin films was flowed and transferred onto Si3N4 substrate and experiment was done in transition mode. The steady-state photoluminescence spectra and time resolved photoluminescence were measured by utilizing Edinburgh luminescence spectrometer (FS 920) and a single photon counting spectrometer (Life Spec II from Edinburgh instruments). The microwave-assisted Stille reaction was working on a CEM DISCOVER instrument.

The PSCs were fabricated in the configuration of ITO/ZnO/PBDB-T :BDT-IC:ITIC/MoO_3/Ag. ITO-coated glass substrates were cleaned sequentially with deionized water, acetone, ethanol, and isopropanol under sonication for 10 min each and then treated with oxygen plasma for 15 min to generate the hydrophilic surface. ETL was prepared by spin coating the ZnO precursor solution (consisting of 0.3 M zinc acetate dihydrate and 0.3 M ethanolamine in 2-propanol) on top of pre-cleaned ITO glass substrates followed by annealing at 120 ^{o}C for 15 min in air. Subsequently, the substrates were transferred into a N_2-filled glove box for spin-coating of the photoactive layer. The active layer (ca. 130 nm) was then spin-coated from the blend solutions (total concentration 16 mg mL^{-1}), which were prepared at 25 ^{o}C in chloroform. The mixed solutions were spin-coated on the top of the ZnO layer

at 1500 rpm for 90 s, followed by thermal annealing at 120 °C for 10 min. Finally, MoO_3 (10 nm) and Ag top electrode were deposited onto the active layer by thermal evaporation at a pressure of 5.0×10-5 Pa. The deposition rate and film thickness were monitored with a quartz crystal sensor. Shadow masks were used to define the active area (4.7 mm^2) of the devices

The current density-voltage (J-V) characteristics were measured using a Keithley 2450 Source-Measure Unit. Oriel Sol3A Class AAA Solar Simulator (model, Newport 94023A) with a 450 W xenon lamp and an air mass (AM) 1.5 filter was used as a light source. The light intensity was calibrated to 100 mW cm-2 by a Newport Oriel 91150V reference cell. The EQE was measured with a solar cell spectral response measurement system (Enli Technology Co., Ltd., QE-R3011). The light intensity was calibrated using a single-crystal Si photovoltaic cell as a standard.

Hole and electron mobility were measured using the space charge limited current (SCLC) method. ITO/PEDOT:PSS/ PBDB-T:BDT-IC/Au for hole-only devices and ITO/ZnO/ PBDB-T:BDT-IC /PDINO/Al for electron-only devices. The SCLC mobilities were calculated by the Mott-Gurney equation:

$$J = \frac{9}{8}\, \mu \varepsilon \varepsilon_0 \,\frac{V^2}{d^3}$$

where J is the current density, εr is the relative dielectric constant of active layer material usually 2-4 for organic semiconductor, herein we use a relative dielectric constant of 4, ε_0 is the permittivity of empty space, μ is the mobility of hole or electron and L is the thickness of the active layer, V is the internal voltage in the device. As shown in Figure S8, S9&S10, the hole and electron mobilities of PBDB-T : BDT-IC (1:1,w/w) are estimated as 6.15×10-4 and 4.59×10-4 cm2 V-1 s -1 respectively, corresponding to nearly balanced charge transport (μ_h/μ_e = 1.03)

Transient absorption data were collected using transient absorption spectroscopy setup. This setup consists of the spectrometer (Ultrafast Helios system) and amplified Ti:Saphhire Laser. The output of amplified Ti:Saphhire Laser provides 800 nm fundamental pulses at 1kHz repetition rate which were splitted into two optical beams to generate pump and probe pulses. One fundamental beam was used to generate pump beam using an optical parametric amplifier (OPA) system (Coherent Opera Solo). A white light/NIR probe was generated by focusing another fundamental beam into a flint glass. Pump and probe beams were focused on a sample and probe light was collected by a charge-coupled device CCD device. The spectral detection region is 0.8 eV to 2.6 eV. The thin film samples were encapsulated using UV curable clue before measurement. The instrument response function (IRF) was ~100 fs FWHM. The samples were excited with the excitation energy 1.62 eV (765 nm) for acceptor excitation and 2.25 eV (550 nm) for donor excitation. The fractional change in transmission was detected in the probe range 0.8-2.6 eV at several time delays.

Note:

The energy loss in our OSCs is calculated using the method proposed by Yao et al. The V_{oc} is determined by the relative proportion of short circuit current and reverse saturation current according to equation S1:[1-4]

$$V_{oc} = \frac{nk_BT}{q}\ln(\frac{J_{sc}}{J_0} + 1) \tag{S1}$$

where n is the ideality factor of the solar cell, k_B is Boltzmann's constant, T is the absolute temperature, q is the elementary charge, J_{sc} is the short circuit current, and J_0 is the reverse saturation current. This formula applies to any recombination mechanism. Considering the specific condition of radiative

recombination, with $J_0 = J_{0,rad}$ and n=1, we can write equation S1 like this:

$$V_{oc,rad} = \frac{k_B T}{q} \ln(\frac{J_{sc}}{J_{0,rad}} + 1) \quad \text{(S2)}$$

The expression for J_{sc} and $J_{0,rad}$ are given by:

$$J_{sc} = q \times \int_0^\infty EQE_{PV}(E)\varphi_{AM1.5}(E)dE \quad \text{(S3)}$$

$$J_0^{rad} = q \times \int_0^\infty EQE_{PV}(E)\varphi_{BB}(E)dE \quad \text{(S4)}$$

Here, EQE_{PV} is the photovoltaic external quantum efficiency, $\varphi_{AM1.5}$ is the standard AM 1.5 solar illumination spectrum, φ_{BB} is the black body emission flux density spectral.

According to Shockley-Queisser (SQ) theory, the photovoltaic external quantum efficiency EQE_{PV}^{SQ} in the SQ limit is defined as:

$$EQE_{PV}^{SQ}(E) = 1, E > E_g;\ EQE_{PV}^{SQ}(E) = 0, E < E_g$$

Then, we can calculate the saturation current density in the SQ limit:

$$J_0^{SQ} = q \times \int_{E_g}^\infty EQE_{PV}^{SQ}(E)\varphi_{BB}(E)dE = q \times \int_{E_g}^\infty \varphi_{BB}(E)dE \quad \text{(S5)}$$

Thus,

$$V_{oc,SQ} = \frac{k_B T}{q} \ln\left(\frac{J_{sc}}{J_0^{SQ}} + 1\right) = \frac{k_B T}{q} \ln(\frac{q \int_0^\infty EQE_{PV}(E)\varphi_{AM1.5}(E)dE}{q \int_{E_g}^\infty \varphi_{BB}(E)dE} + 1) \quad \text{(S6)}$$

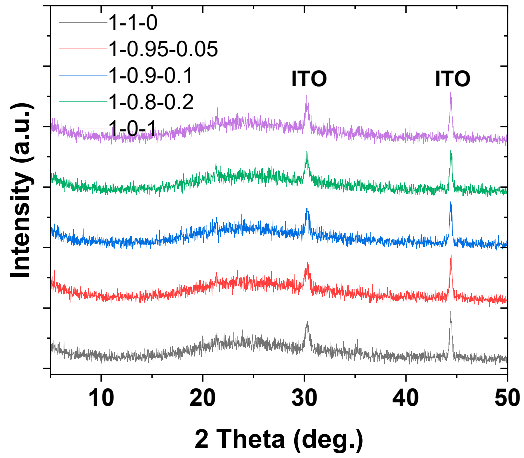

Figure S1 The resultant XRD patterns of active layers with various compositional ratios.

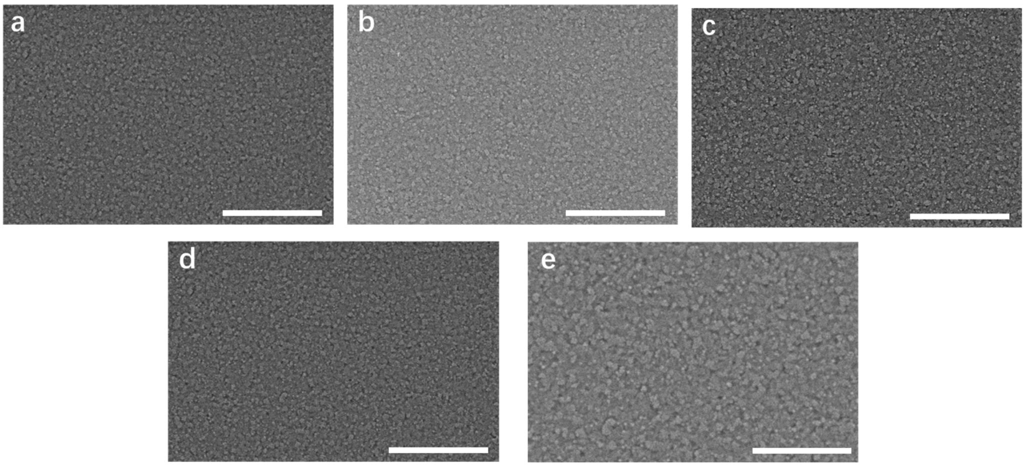

Figure S2 SEM images of organic active layers of 1-1-0 (a), 1-0.95-0.05 (b), 1.-0.9-0.1 (c), 1-0.8-0.2 (d) and 1-0-1 (e).

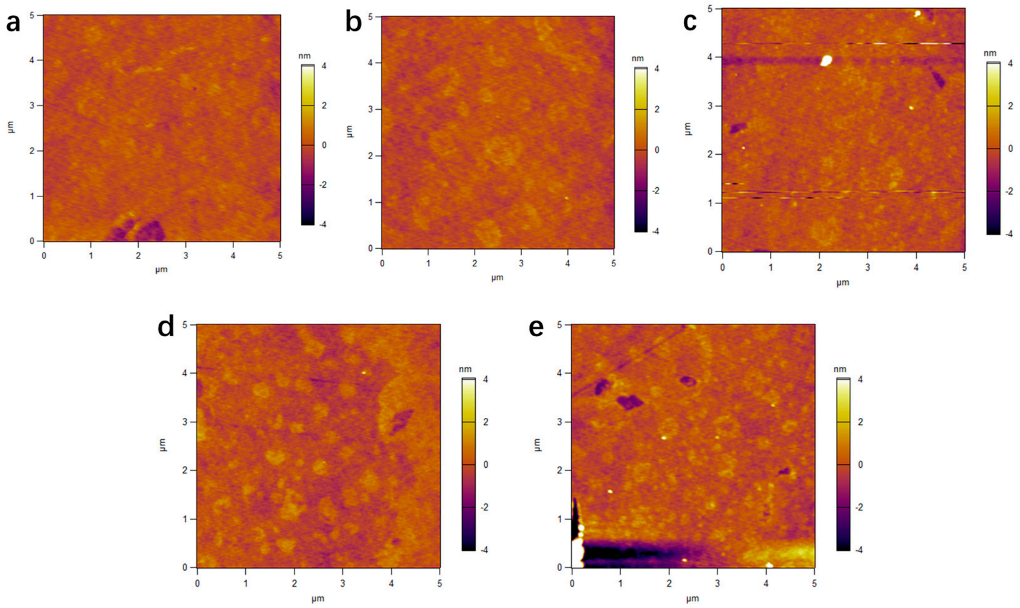

Figure S3 AFM images of organic active layers of 1-1-0 (a), 1-0.95-0.05 (b), 1.-0.9-0.1 (c), 1-0.8-0.2 (d) and 1-0-1 (e).

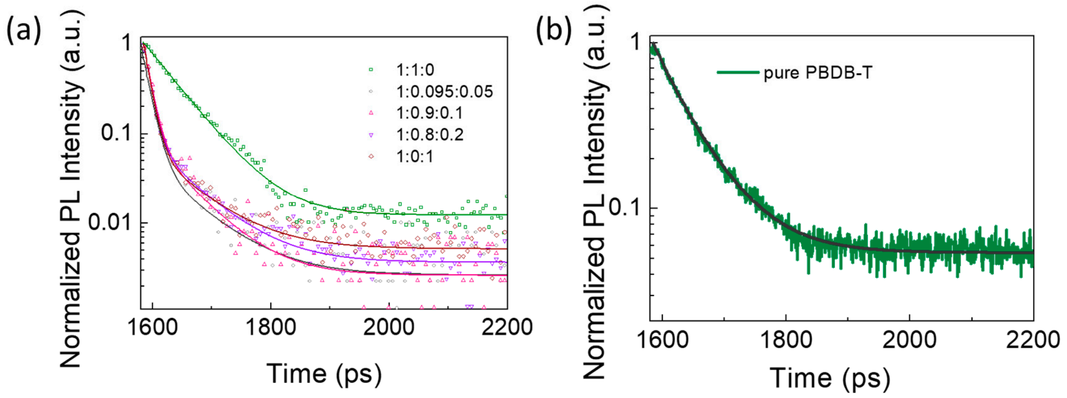

Figure S4. (a) Time resolved fluorescence of blend system with different BDT-IC ratio. (b) Time resolved fluorescence of pure PBDB-T**.**

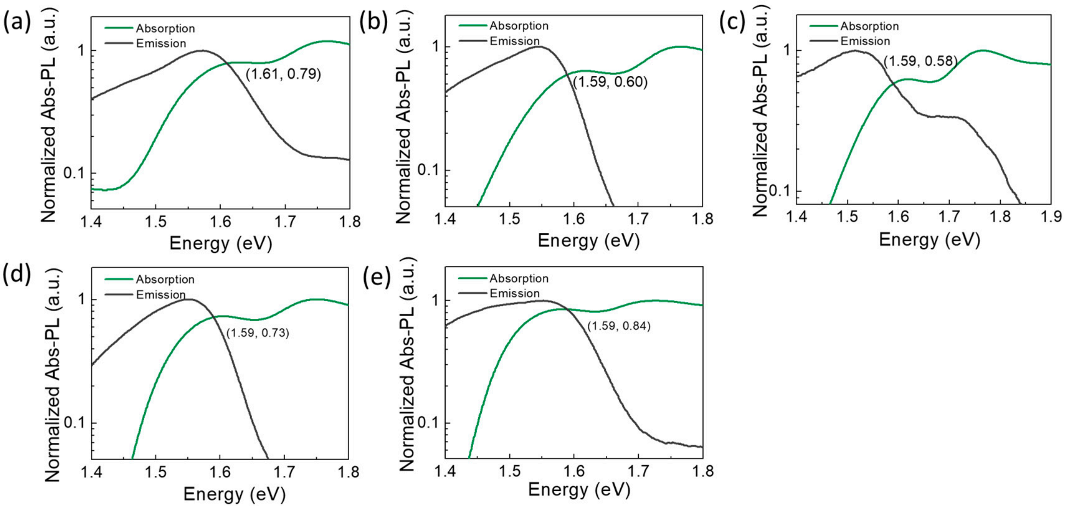

Figure S5. Normalized absorbance and photoluminescene spectra based on PBDB-T:ITIC-TH :BDT-IC with different weight ratio (a) (1:1:0, w:w:w) (b) (1:0.95:0.5, w:w:w) (c) (1:0.9:0.1, w:w:w) (d) (1:0.8:0.2, w:w:w) (e) (1:0:1, w:w:w).

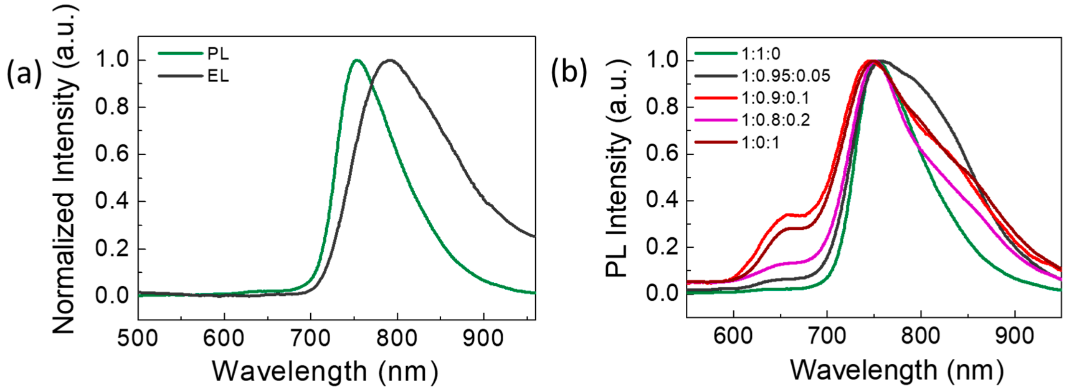

Figure S6. (a) PL and EL curves of PBDB:ITIC-TH. (b) PL spectra with different weigh ratio of BDT-IC.

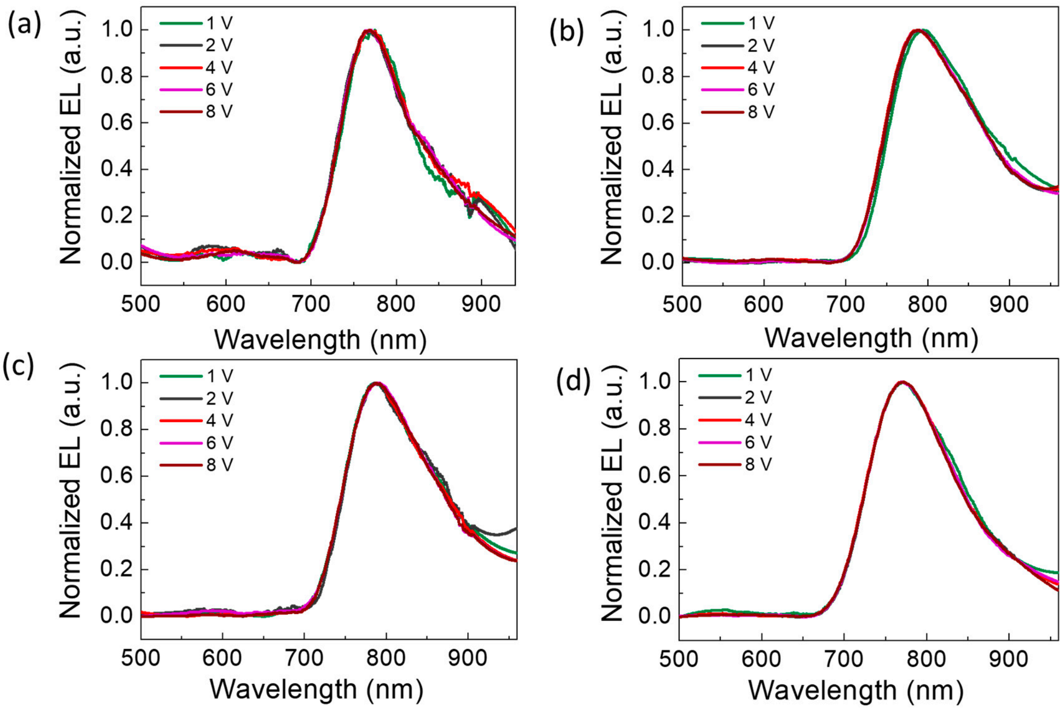

Figure S7. EL spectra of pure ITIC-TH and PBDB-T:ITIC-TH, PBDB-T:ITIC-TH:BDT-IC (1:0.9:0.1), PBDB-T:BDT-IC blend system under diverse forward biases.

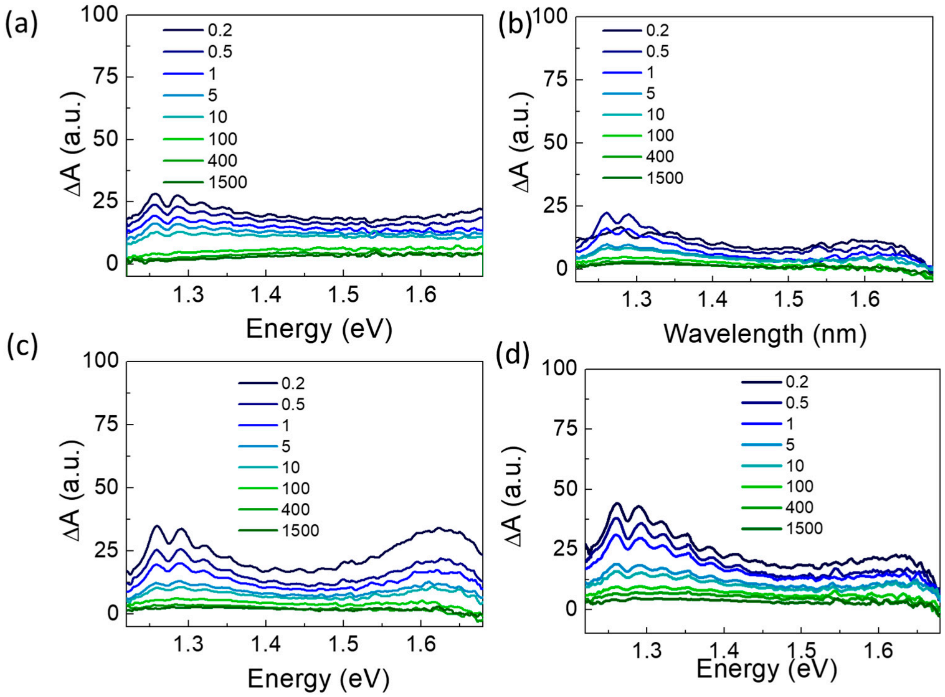

Figure S8. Transient absorption spectra of (a) pristine PBDB-T film (b) blend film PBDB-T:ITIC-TH:BDT-IC (1:1:0, w:w:w) (c) PBDB-T:ITIC-TH:BDT-IC (1:0.9:0.1, w:w:w) (d) PBDB-T:ITIC-TH:BDT-IC (1:0:1, w:w:w) at 3.1 eV excitation and different time (ps) in the near infrared region.

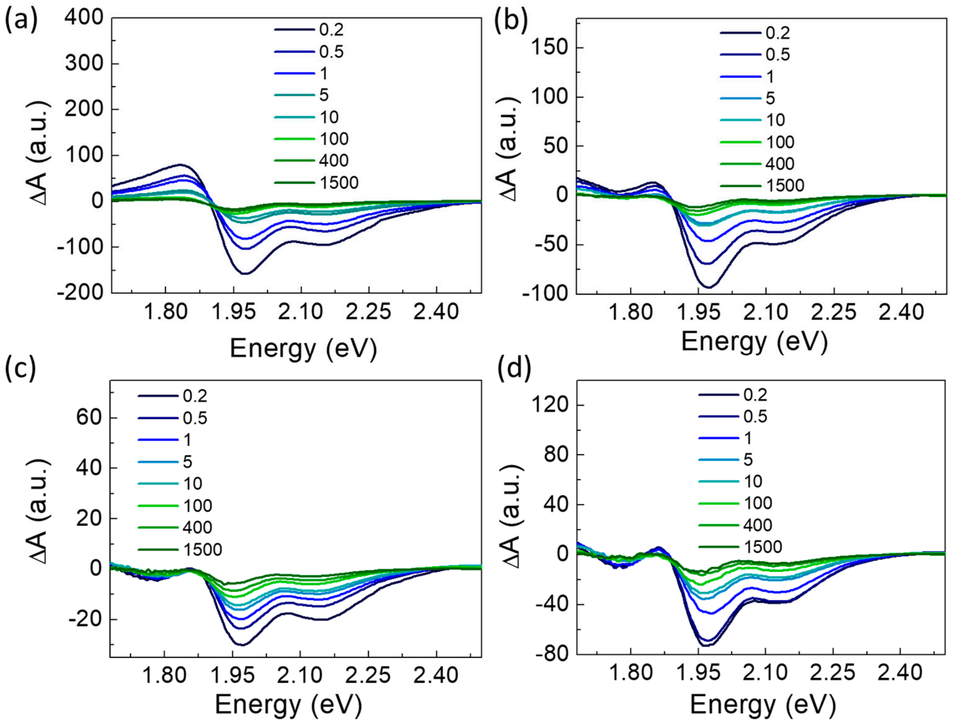

Figure S9. Transient absorption spectra of (a) pristine PBDB-T film (b)blend film PBDB-T:ITIC-TH:BDT-IC (1:1:0, w:w:w) (c) PBDB-T:ITIC-TH:BDT-IC (1:0.9:0.1, w:w:w) (d) PBDB-T:ITIC-TH:BDT-IC (1:0:1, w:w:w) at 3.1 eV excitation and different time (ps) in the visible light region.

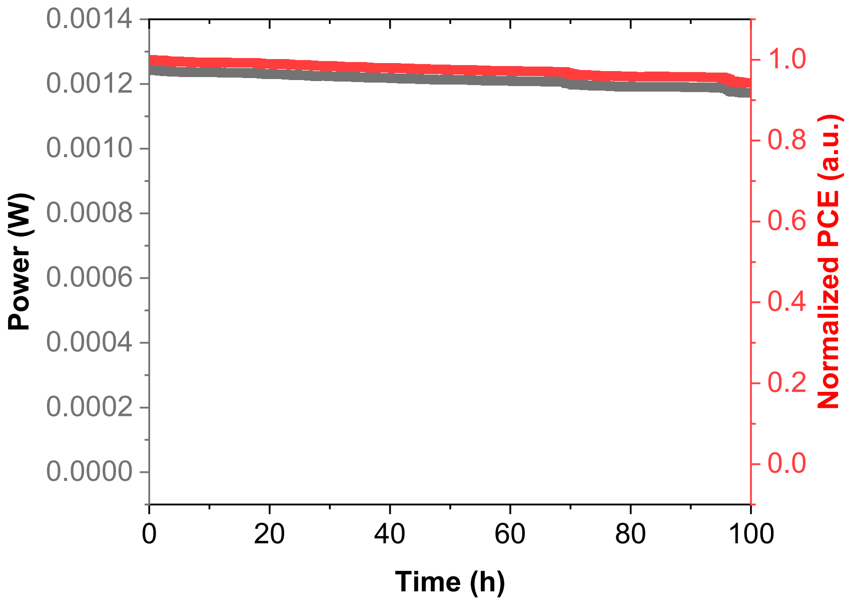

Figure S10. Maximum power point tracking (MPPT) measurements of optimal PV device at 25 °C.

Table S1. PLQY of solid films based on PBDB-T, ITIC-TH and BDT-IC or blended system.

**Films**	**PLQY (%)**
PBDB-T	2.38
ITIC-TH	1.17
BDT-IC	4.00
1:1:0	0.58
1:0.95:0.05	0.45
1:0.9:0.1	0.06
1:0.8:0.2	0.35
1:0:1	0.36

Table S2. PL life time parameters and quenching efficiency obtained from a bi-exponential fitting of time resolved data. τ_p represent the PL lifetime of pristine PBDB-T film. $\tau_{D:A}$ stand the PL lifetime of blend films. $\emptyset_t$ is the PL quenching efficiency obtained from transient PL.

**Active layer**	$\boldsymbol{\tau_p \times 10^{-9}}$ **s**	$\boldsymbol{\tau_{D:A} \times 10^{-10}}$ **s**	$\boldsymbol{\emptyset_t = 1 - {}^{1/\tau_p}\!/_{(1/\tau_p + 1/\tau_{D:A})}}$	**ΔPL**
**Pure PBDB-T**	3.118	-	-	-
1:1:0	-	30.35	50.6%	43%
1:0.95:0.05	-	8.265	79.0%	53%
1:0.9:0.1	-	7.979	79.6%	83%
1:0.8:0.2	-	8.448	78.8%	73%
1:0:1	-	8.470	78.6%	61%

REFERENCES

(1) Tang, A.; Xiao, B.; Wang, Y.; Gao, F.; Tajima, K.; Bin, H.; Zhang, Z.-G.; Li, Y.; Wei, Z.; Zhou, E. Simultaneously Achieved High Open-Circuit Voltage and Efficient Charge Generation by Fine-Tuning Charge-Transfer Driving Force in Nonfullerene Polymer Solar Cells. *Advanced Functional Materials* **2018,** *28* (6), 1704507, DOI: 10.1002/adfm.201704507.

(2) Qian, D.; Zheng, Z.; Yao, H.; Tress, W.; Hopper, T. R.; Chen, S.; Li, S.; Liu, J.; Chen, S.; Zhang, J.; Liu, X. K.; Gao, B.; Ouyang, L.; Jin, Y.; Pozina, G.; Buyanova, I. A.; Chen, W. M.; Inganas, O.; Coropceanu, V.; Bredas, J. L.; Yan, H.; Hou, J.; Zhang, F.; Bakulin, A. A.; Gao, F. Design rules for minimizing voltage losses in high-efficiency organic solar cells. *Nat Mater* **2018,** *17* (8), 703-709, DOI: 10.1038/s41563-018-0128-z.

(3) Liu, J.; Chen, S.; Qian, D.; Gautam, B.; Yang, G.; Zhao, J.; Bergqvist, J.; Zhang, F.; Ma, W.; Ade, H.; Inganäs, O.; Gundogdu, K.; Gao, F.; Yan, H. Fast charge separation in a non-fullerene organic solar cell with a small driving force. *Nature Energy* **2016,** *1* (7), DOI: 10.1038/nenergy.2016.89.

(4) Eisner, F. D.; Azzouzi, M.; Fei, Z.; Hou, X.; Anthopoulos, T. D.; Dennis, T. J. S.; Heeney, M.; Nelson, J. Hybridization of Local Exciton and Charge-Transfer States Reduces Nonradiative Voltage Losses in Organic Solar Cells. *J Am Chem Soc* **2019,** *141* (15), 6362-6374, DOI: 10.1021/jacs.9b01465.
